# Systematic reanalysis of genomic data by diagnostic laboratories: a scoping review of ethical, economic, legal and (psycho)social implications

**DOI:** 10.1038/s41431-023-01529-z

**Published:** 2024-03-14

**Authors:** Marije A. van der Geest, Els L. M. Maeckelberghe, Marielle E. van Gijn, Anneke M. Lucassen, Morris A. Swertz, Irene M. van Langen, Mirjam Plantinga

**Affiliations:** 1 Department of Genetics, University Medical Center Groningen, University of Groningen, Groningen, The Netherlands; 2 Institute for Medical Education, University Medical Center Groningen, University of Groningen, Groningen, The Netherlands; 3 Faculty of Medicine, Clinical Ethics and Law, University of Southampton, Southampton, UK; 4 Centre for Personalised Medicine, Wellcome Centre for Human Genetics, Nuffield Department of Medicine, University of Oxford, Oxford, UK

**Keywords:** Medical genetics, Medical ethics

## Abstract

With the introduction of Next Generation Sequencing (NGS) techniques increasing numbers of disease-associated variants are being identified. This ongoing progress might lead to diagnoses in formerly undiagnosed patients and novel insights in already solved cases. Therefore, many studies suggest introducing systematic reanalysis of NGS data in routine diagnostics. Introduction will, however, also have ethical, economic, legal and (psycho)social (ELSI) implications that Genetic Health Professionals (GHPs) from laboratories should consider before possible implementation of systematic reanalysis. To get a first impression we performed a scoping literature review. Our findings show that for the vast majority of included articles ELSI aspects were not mentioned as such. However, often these issues were raised implicitly. In total, we identified nine ELSI aspects, such as (perceived) professional responsibilities, implications for consent and cost-effectiveness. The identified ELSI aspects brought forward necessary trade-offs for GHPs to consciously take into account when considering responsible implementation of systematic reanalysis of NGS data in routine diagnostics, balancing the various strains on their laboratories and personnel while creating optimal results for new and former patients. Some important aspects are not well explored yet. For example, our study shows GHPs see the values of systematic reanalysis but also experience barriers, often mentioned as being practical or financial only, but in fact also being ethical or psychosocial. Engagement of these GHPs in further research on ELSI aspects is important for sustainable implementation.

## Introduction

Next Generation Sequencing (NGS) techniques are already widely used in genetic research and diagnostics. However, continuous improvement in these approaches is leading to the identification of a rapidly growing number of disease-associated variants [1, 2]. In 2022, almost 13,000 new classifications were added to the Dutch VKGL variant classification database [3], demonstrating the enormous rate of new discoveries that produce new genetic information.

In some cases, a patient’s NGS data is reanalyzed in response to a new diagnostic request. This is usually initiated by the clinician, or even by the patient, for example when they have a family member for whom new information was found [1]. Due to the ongoing progress of identifying novel gene-disease associations in genetics, more and more cases exist of patients for whom it was not possible to determine a diagnosis at the time of initial genetic testing, but with new insights a suitable diagnosis might be found at a later timepoint. One example of this scenario was highlighted by Wenger et al. [4]. A clinical laboratory issued a nondiagnostic exome report for a young female with severe developmental and neurological symptoms. After whole-exome sequencing (WES) analysis, no genetic diagnosis could be made. However, just two weeks later, a study was published linking a variant present de novo in the patient’s exome to a syndrome that fit the patient’s phenotype. Only after reanalysis incorporating the new information was this patient correctly diagnosed. This illustrates how reappraisal of existing data might explain many previously unsolved cases.

Usually, reanalysis is only conducted reactively, upon request, when new information becomes available, however, the Wenger et al. [4] case, as well as other examples, show that more proactive reanalysis might be very effective, potentially increasing diagnostic yield by up to 10% [4]. The first results of the Solve-RD initiative—a project in which diagnostic centers all over Europe have joined forces to solve rare disease cases using data reanalysis approaches—show equally promising results [5]. Increasing the number of (specific) diagnoses can lead to new or better-fitting treatment and to more effective counseling for patients and families. For this reason, many studies now suggest that *systematic* reanalysis of NGS data should be introduced into routine diagnostics [6–9]. In a systematic approach, reanalysis is not initiated at the request of the clinician or patient but is rather done repeatedly by the clinical laboratory for previously undiagnosed patients, whether or not there is an indication of new information. This would mean that the laboratory reanalyzes the patient’s previously sequenced raw data to look for all the genes and variants currently proven to be associated with the patient’s condition, including genes that were not previously analyzed because they had not been connected with the patient’s condition at the time of the original analysis [10].

In 2017, O’Daniel et al. [11]. Reported that most laboratories did not have any policies regarding routine data reanalysis. In 2019, the American College of Medical Genetics and Genomics published a statement comprising practical points for laboratories to consider regarding reanalysis [12]. Introducing systematic reanalysis not only has practical implications for laboratories, it also brings ethical, economic, legal and (psycho)social (ELSI) aspects into play. For example, reanalysis might raise a dilemma about whether already scarce time and personnel should be invested in reanalysis, or if this can be better used for new patients only [13]. Furthermore, reanalysis can become a resource-intensive task, for which (semi-)automated approaches, such as the application of machine learning techniques, should be explored that will probably introduce even more ELSI considerations.

To identify these and other ELSI aspects of systematic reanalysis, we performed a scoping literature search and subsequent review. With this review, we aim to advise clinical laboratories considering the introduction of systematic reanalysis with NGS data on the ELSI aspects they should be taking into account.

## Materials and methods

### Definitions

During our research, we identified several ways of reevaluating NGS data from the literature. For clarity, we use the definitions described by El Mecky et al. and Carrieri et al. [10, 14]. In this context, *reanalysis* is defined as reviewing existing raw NGS data for a patient, including all variants and genes not previously linked to the patient’s phenotype. *Reinterpretation* is defined as reviewing existing variants that were previously linked to the patient’s disease/phenotype to assess whether these variants still have the correct interpretation, or if the variant(s) should be reclassified based on new knowledge. *Reclassification* is defined as assigning a different classification to an already known variant, based on new knowledge, as a result of reinterpretation. For example, the reclassification of a variant of uncertain significance (VUS) to (likely) pathogenic. *Retesting* is defined as ordering a new genetic test, thus generating new data [10, 14].

The term ‘ELSI’—Ethical, Legal and Social Issues—originates from the Human Genome Project, which was the first project for which an ELSI program was established to address and approach these issues in order to develop guidelines and policy on practices concerning human genetics [15]. Since then, ELSI research has developed into a full multidisciplinary research field, and several other aspects have been added to the area of interest, e.g., psychosocial and economic aspects.

### Literature search strategy

We systematically searched the literature using PubMed. We also searched Google Scholar, but this did not result in inclusion of any additional studies. In our search strategy, we searched in the title and abstract for the MeSH terms ‘Genetics’ or ‘High-Throughput Nucleotide Sequencing’ or one of the following synonyms: ‘Sequence data’, ‘Exome sequencing’, ‘NGS’ or ‘WES’. These terms were combined with ‘reanalysis’ or ‘re-analysis’. We deliberately eliminated search terms restricted to ELSI in our initial search, as this might exclude relevant articles that do not have an ELSI focus but do mention relevant ELSI aspects. Furthermore, we excluded literature from before 2008 as NGS had not yet been implemented in diagnostic settings at that time.

### Selection of papers

**Fig. 1:**
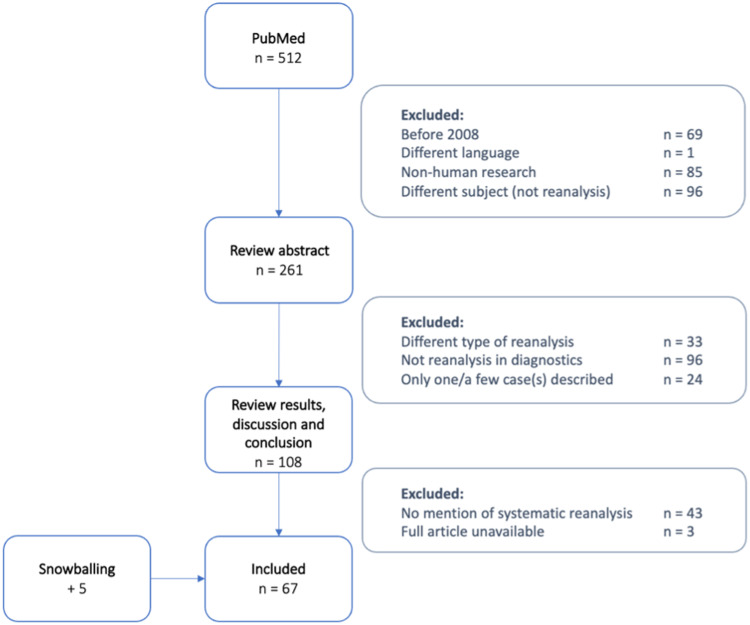
Flow-chart of the article selection process. Literature search results for systematic reanalysis in genetic diagnostics.

Figure 1 shows the process for selection of papers. Our search terms resulted in 512 papers found through PubMed. We then reviewed these articles based on their metadata and title. In total, we excluded 251 articles because they (i) were published before 2008, (ii) were not available in English, (iii) concerned non-human research, or (iv) were about a different subject or field. After reviewing the abstracts, we excluded another 153 articles because these studies had either conducted a different type of reanalysis, such as reanalysis of pedigrees, or had no application in diagnostics. Finally, we excluded 46 articles because they did not mention systematic reanalysis at all. Via a snowballing approach, we were able to include another five articles, leading to inclusion of 67 articles in the study.

### Analysis

First, open coding was performed by scanning the articles and selecting all the information that was mentioned in the context of (systematic) reanalysis. Secondly, axial coding was done to identify the most common themes and to cluster them into relevant concepts. After coding, seven unique concepts were identified and categorized as ethical (E), economic (EC), legal (L), or (psycho)social (PS) aspects. In some cases, the concepts appertain to different categories, as shown in Table 1. Finally, we also identified practical (P) aspects.

**Table 1:** Concepts related to ELSI aspects identified by open coding.

ELSI Aspect	Related concepts identified in the literature
Practical	Approach to reanalysis
	Diagnostic yield
	Intervals
Ethical	Duty & responsibility
	Consent
Legal	Duty & responsibility
	Consent
(Psycho)social	Perceptions of professionals toward reanalysis
	Communication (of results)
	Time investment/workload
economic	Time investment/ workload
	Costs & cost-effectiveness

## Results

### Characteristics of the articles

**Fig. 2:**
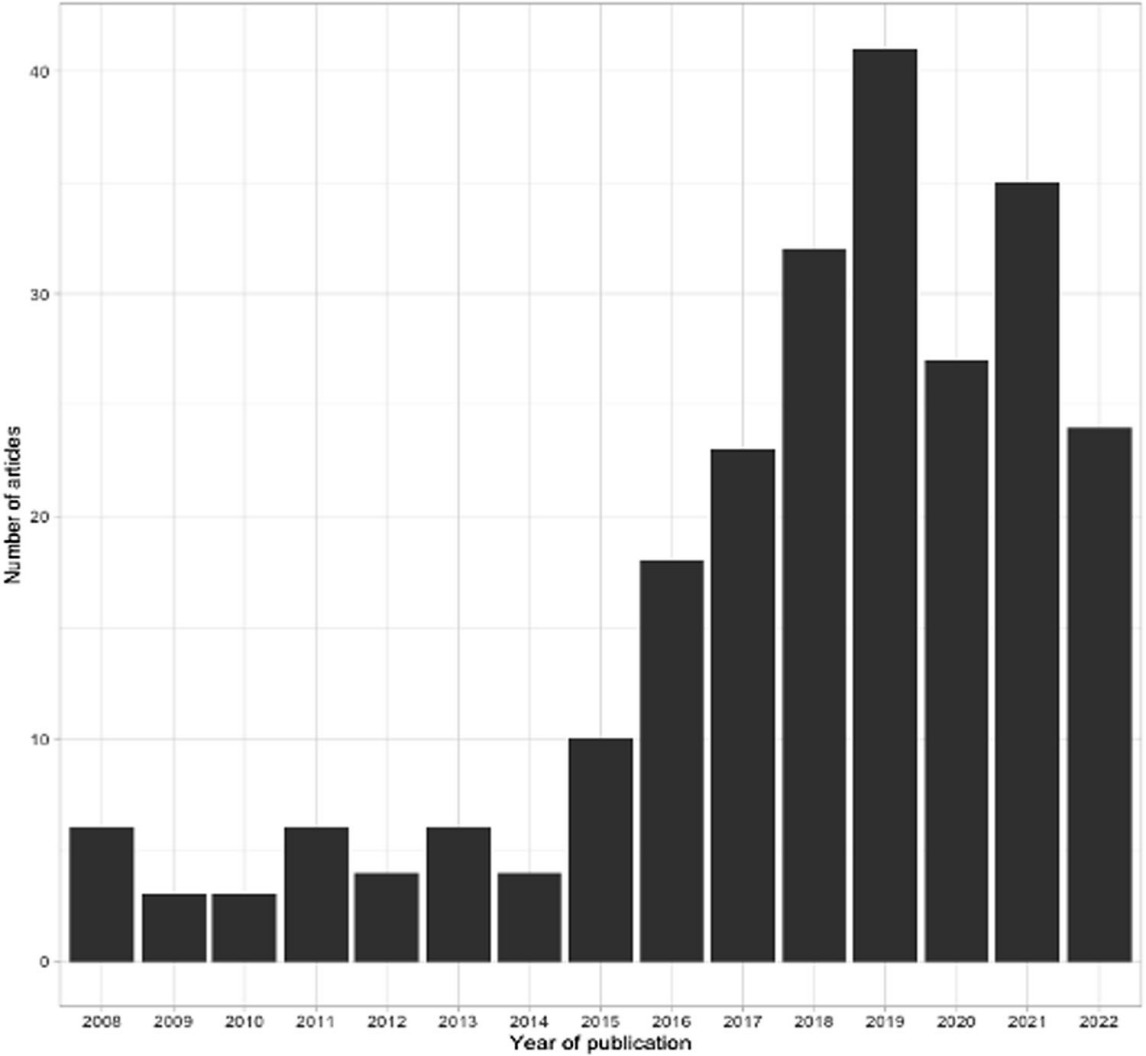
Increase in literature covering reanalysis. Number of publications about reanalysis over time, from 2008 to 2022.

From all the articles for which we screened abstracts (*N* = 261, see Fig. 1), it is clear that the scientific literature covering reanalysis has increased greatly over time (see Fig. 2). However, most articles focus on the practical aspects of reanalysis and only implicitly touch upon classical ELSI aspects. In total, we included 67 articles in this literature review. An overview and the concepts identified per article are shown in Supplementary Table 1. The oldest paper included is relatively recent (2016), which can be explained by the recent implementation of NGS techniques in diagnostics. Furthermore, most articles are by authors based in the USA.

Our research found that there is linguistic ambiguity around the term *reanalysis*, with different terms used interchangeably in the included literature. *Reevaluation* is often used as a synonym for *reanalysis* [4, 13, 16–19], while reanalysis is also used as a synonym for *reinterpretation* [20, 21]. Often, the definition of reanalysis used is unclear [11, 22–26]. In one case, reanalysis was used interchangeably with reinterpretation [27]. In other cases, reanalysis is defined similarly to the previously described definitions of variant reinterpretation or *retesting* [28–30].

### Motives and approaches for pursuing systematic reanalysis (practical)

Most articles describe an increase in the expected diagnostic yield as the main reason to consider implementation of systematic reanalysis. Almost all the studies that conducted reanalysis reported an increase in diagnostic yield [4, 11, 17, 20, 21, 24–28, 30–51], but the increase varied widely between the studies. One possible explanation for this variation is differences in the analytical or technical approaches used, which make it difficult to compare studies. Differences between studies include (i) differences in the type of NGS data used (whole-genome sequencing (WGS) or WES), (ii) differences in the time after initial analysis when reanalysis was performed, (iii) differing sample sizes, (iv) differences in population selection, (v) whether trio sequencing was (or could be) performed or the study was proband-only, (vi) differences in the approach to reanalysis (manual or (semi-)automated) and (vii) differences in the way of reporting results (increase relative to the complete initial sample size or only to previously undiagnosed cases).

Overall, the reported increase in diagnostic yield compared to initial total sample set ranged from 6.5% [25] to 41% [8]. These differences in yield can mainly be explained by differing approaches to reanalysis. The studies that reported a higher diagnostic yield had performed comprehensive manual reanalysis in which they either specifically selected likely Mendelian cases for reanalysis, used trio sequencing or had a smaller sample size, [52, 53], whereas studies that reported lower diagnostic yield had larger sample sizes and performed largely automated reanalysis [25, 30].

One of the main ELSI-related questions raised in the literature was at what intervals systematic reanalysis should be initiated after the first analysis. Recommendations from empirical studies on this topic ranged from 6 months [37, 38] to 3 years [4]. Li et al. [37] recommend at least 6 months, as they observed a large increase in diagnostic yield by reanalysis after >6 months compared to <6 months. Although not statistically significant, another study discussed that an interval of at least 12 months would be preferrable since it would increase the chance of new relevant clinical information being available [32]. Regarding optimal intervals for reanalysis, Stark et al. made another point [54]. Their cost-effectiveness analysis showed that WES data reanalysis at 18 months was more cost-effective than reanalysis at 6 months, at least for expected Mendelian diseases.

Furthermore, where some articles assert that retesting with WGS might be more efficient than reanalyzing WES data, as WGS provides more information [55] Hiraide et al. [56] argue “It is important to reanalyze WES data before additional testing, such as whole-genome sequencing” as the increase in diagnostic yield from WES reanalysis is already high.

### ELSI aspects

#### Duty and responsibilities (ethical, legal)

All the articles that addressed duty and responsibility stated there is currently no legal duty for clinical laboratories to reanalyze data [11, 22, 23, 57, 58]. However, Vears et al. [23, 58] argued that the added value of reanalysis is of such importance, as the increase in diagnostic yield might result in important changes in the treatment, that laboratories have good reasons to incorporate it in routine diagnostics. In response, other authors have suggested that many labs will not have the resources to adequately implement systematic reanalysis without losing focus on other tasks, while reanalysis on request is already often done [11]. The current consensus is therefore that there should be no (legal) duty, in general, for laboratories to perform reanalysis [23, 57]. However, Mensah et al. [59] suggest that a perceived responsibility might shift toward a duty as more automated approaches become available, thereby making reanalysis more feasible. They argue the duty would exist for clinical laboratories as they are responsible for data storage and analysis, and that a legal precedent could be set to make laboratories liable when not adopting reanalysis.

All the articles that stated there is not yet a duty to reanalyze referred to a statement made by EuroGentest – a European network that aims to harmonize genetic testing services - (statement 5.05): “The laboratory is not expected to reanalyze old data systematically and report novel findings, not even when the core disease genes panel changes” [60]. The authors add that the patient is responsible for contacting the clinician to initiate reanalysis, and the laboratory should not be made responsible for reanalyzing all *raw* data. However, they also state that when a variant is reclassified, the laboratory is responsible for reissuing the diagnostic report and even for recontacting the patient. Furthermore, they add that a diagnostic request is *“*a contract at a certain point in time*”*. In other words, when the report is completed, whether it was a diagnostic report or not, there is no further responsibility or duty for the laboratory to pursue finding a diagnosis. Potential liabilities are not described in literature, with only Mensah et al. [59] mentioning leaving patients without a diagnosis as a potential liability of not periodically pursuing reanalysis.

Although there is currently no duty for clinical laboratories to (systematically) reanalyze NGS data, it has been argued that systematic reanalysis initiated by the laboratory would be the ideal approach [58]. Ji et al. [61] highlight a responsibility for the clinical laboratory to recontact the patient and clinician when new information comes to light, based on the principle of *beneficence* or ‘doing good’. Another argument they mention is that putting the responsibility in the hands of the laboratory would remove a step in the process in that the clinician would not have to go back to the laboratory and thereby also avoiding the risk that patients might forget to initiate reanalysis or might not be aware of the urgency. Other corresponding responsibilities for the laboratory in effectively and systematically reanalyzing data would be issuing the diagnostic report and contacting the referring clinician [57], ensuring data preservation and remaining up-to-date about new findings [13].

#### Consent (ethical, legal)

An ethical and legal aspect found in the literature was *consent*. Here we distinguish between the consent form—a written and signed legal document—and the broader consent process. A study by Vears et al. [22] showed that many consent forms regarding NGS in diagnostics do not currently mention the possibility of recontacting the patient and providing updated information at a later point in time. If mentioned, there are differences in how much information about the potential new insights is given to the patient.

If systematic reanalysis initiated by the laboratory is to be implemented, the consent processes need to be changed to include more information, both in counseling and on forms, about the possibility to be contacted with new information [19, 58, 62, 63]. Appelbaum et al. [13] and Deignan et al. [12] emphasized that patients in pre-test counseling should be made aware of possibly uncertain test results and the possibility of reanalysis, as well as of the opportunity to opt out of future reanalysis. These authors expect, however, that the latter option will not be used intensively as they assume that patients want the most complete and accurate interpretation of results. However, in a study by Fung et al. [41], 2 out of 61 families recontacted for reanalysis refused to participate, e.g., because the condition improved and they did not see the need for further reanalysis. Appelbaum et al. [13] argue that when systematic reanalysis becomes a possibility and is included in the consent and counseling process, ex-patients who did not have this option in the past should still be given the opportunity to be included in reanalysis, requiring an update of consent from previous patients as well.

#### Professional’s perceptions toward reanalysis (psychosocial)

Another issue and (psycho)social concept is *perceptions of professionals toward systematic reanalysis*. Little is discussed about the current perceptions and related dilemmas of laboratory professionals in general. Only two papers assessed this topic [14, 58]. El Mecky et al. [14] conducted focus groups with Dutch academic clinical laboratory geneticists. Although their study did not focus solely on reanalysis, some participants briefly mentioned that they believe systematic reanalysis might be an effective approach for undiagnosed cases. However, they also stated that retesting might be more effective. Furthermore, the participants stressed the importance of being supplied with optimal and updated information regarding the phenotypes of both patients and their families, information that is currently regarded as too brief and unsystematic as there are no digital systems in place to facilitate this type of contact between the laboratory and clinicians or patients.

In interviews with genetic health professionals (GHPs) regarding initiating reanalysis conducted by Vears et al. [58], a laboratory-initiated model for systematic or routine reanalysis was discussed. Participants indicated that this model would be interesting because it would remove a step from the process as the initial communication between laboratory and clinic is omitted and the request for reanalysis by the clinic does not have to be scheduled as it is already part of the routine process. But even the participants who preferred laboratory-initiated systematic reanalysis still acknowledged that it may not be feasible due to a current lack of resources and funding [58]. In contrast, Segal et al. [64] propose a clinician-centered model, including an automated platform, to incorporate reanalysis as part of follow-up visits. They state that a laboratory-initiated model would be difficult to implement considering the limited updated information they currently receive from the clinician. However, regardless of the preferred model, multiple clinical geneticists stated that both the laboratory and the clinic feel a responsibility for regular reanalysis, and both would like to be able to provide it as a service.

#### Communication (of results) (psychosocial)

Zastrow et al. [29] emphasize that iterative communication between the clinical laboratory, referring clinician and possibly the clinical geneticist is a very important factor in the success of interpretation of (new) results. Al-Murshedi et al. [7] also stress that (exome) reports should be carefully evaluated by a GHP to make sure that the potential disease-causing variant reported does account for the patient’s current medical situation. However, there are complicating factors for adequate communication. One question is whether all the information found should be reported back to the referring clinician, or if only clinically relevant findings according to the laboratory are sufficient considering the additional workload for GHPs and other clinicians compared to the expected clinical yield [13].

According to Neu et al. [18] iterative communication between the laboratory, the geneticist and the referring specialist would be an ideal way to come to optimal results. However, this would be very labor-intensive. As a solution, Basel-Salmon et al. [24] mention the need for digital tools that enhance rapid and in-depth communication, for example on phenotypic details, in a standardized manner (such as HPO terms). Sarmady and Tayoun [52] also mention an environment of interaction, but they add that the potential knowledge gaps between laboratory personnel (clinical literacy) and clinicians (genetic literacy) need to be considered.

#### Time investment and workload (psychosocial, economical)

In addition to the approaches to reanalysis described and the effects on diagnostic yield, concerns about workload are also mentioned in the literature. A less automated reanalysis process will be more labor-intensive, which can strain qualified personnel. This burden on personnel is also one of the main points of concern about implementing systematic reanalysis [16, 17, 31, 40, 54, 63, 65–67]. In particular, the time needed for variant interpretation can become a large burden [27, 42]. To reduce workload, several bioinformatic tools are already being developed, e.g., tools using machine learning approaches to partially automate reanalysis [17, 40, 59, 68, 69]. However, O’Brien et al. [69] mention that, although their tool decreases analysis time, human input is still required, at least to decide whether the information found should be reported.

Smith et al. [70] bring in a different perspective. They hypothesize that it could be more time-efficient and thorough to systematically review all undiagnosed cases with rare variants in genes for which new evidence becomes available, rather than performing reanalysis only upon request. In line with this, Sarmady and Tayoun [52] propose an efficient model for ongoing reanalysis in which reanalysis is only triggered when new, potentially relevant, information becomes available in online databases. In another option mentioned by Hiatt et al. [6], when reanalysis of all generated data is not considered feasible, automated flagging of variant–gene combinations newly linked to a phenotype can provide a lot of information.

#### Costs and Cost-effectiveness (economical)

In addition to the previously mentioned cost-effectiveness analysis by Stark et al. [54], two similar studies by Ewans et al. [32, 55] showed that WGS resulted in the best diagnostic yield for Mendelian disorders, but WES with systematic reanalysis is more cost-effective if a small reduction in diagnostic yield is acceptable. Furthermore, an analysis of patients with pediatric-onset diseases by Fung et al. [41] showed a minimum savings of €17,282 in healthcare costs per individual, with reanalysis leading to a long-term change in clinical outcome and subsequently routine management.

Finally, another consideration mentioned in literature is the option of systematic genetic retesting compared to reanalysis. Some articles mention that the data storage costs and burden can become too large, making ordering a completely new test (e.g., resequencing of already available DNA samples) more interesting because the costs for sequencing are still decreasing [31, 33]. This option was also mentioned in the focus groups conducted by El Mecky et al. [14].

## Discussion

### Summary of the main results

In this literature review we explored the current literature on the ethical, economic, legal and (psycho)social implications of implementing systematic reanalysis of NGS data, from the perspective of clinical laboratories. Interestingly, our results show that it is mainly ELSI-related practical aspects, issues and (potential) solutions that are addressed in literature, with no explicit recognition and mention of the inherent ELSI implications that need to be considered for responsible implementation. Many papers suggest that systematic reanalysis should be implemented in routine diagnostics, citing an increase in diagnostic yield as the main justification, but they do not describe the potential trade-offs that would have to be made in this process. However, implementation has not yet started. The most common concern regarding implementation of systematic reanalysis is the fear of increasing workloads and costs. Such seemingly practical issues also often have implicit ELSI aspects regarding duties and responsibilities, consent, professional perceptions, communication, and associated costs, and we discuss these issues further in the next paragraphs.

Existing ambiguities about the definitions of reanalysis and related terms make it difficult to extract information about specific ELSI aspects from literature. Therefore, we further specify these definitions in Table 2, based on their current use in literature and previously described definitions by our group [14].

**Table 2:** Specification of definitions for reanalysis and related terms.

Reevaluation	An overarching term describing the consideration of revisiting the patient’s information, without specifying which approach will be taken.
Retesting	The process in which the patient’s sample is tested again using the same or a different technique (also known as resequencing), resulting in a new set of raw data. For example, WES can be performed again using updated approaches, or WGS can also be performed, resulting in new raw data.
Reanalysis	Using the patient’s existing raw data, sequenced in the past, to analyze all genes currently associated with the patient’s condition (including genes not analyzed previously).
Reinterpretation	Reinterpretation of genetic variants that have been detected, analyzed and interpreted in the past to assess whether the initial classification is still correct or should be changed in light of new information. In contrast to reanalysis, reinterpretation only applies to the genes and variants that were known to be associated with the patient’s condition at the time of first referral. New genes or variants are not taken into account.
Reclassification	The action in which a variant receives a new classification, as a result of reinterpretation, in regard to the classification system described by Richards et al.

### ELSI considerations and systematic reanalysis

First, the papers that mentioned duty unanimously agree that there is currently no legal duty to (systematically) reanalyze, based on a statement made by EuroGentest. Although EuroGentest is an authority in guidance on genetic testing, some remarks can be made regarding this statement. First, the statement was made in 2014, and the field and the techniques used have evolved since that time, with NGS becoming far more embedded in diagnostics. In addition, the EuroGentest comment that the laboratory has a responsibility to reissue a report when new information becomes available contradicts their argument that a diagnostic request is a contract at a specific point in time, as reinterpretation also happens after the first report was issued.

The PHG foundation, a non-profit think tank affiliated with the University of Cambridge, also criticized the EuroGentest statement and requested a revision in 2014 [71]. They commented that if there is no prospective (systematic) reanalysis, the timing of the test would become a determinant for test outcomes, giving currently undiagnosed patients a lower quality of care compared to future patients, resulting in inequity. Although this is almost always the case in healthcare, two important remarks can be made for the field of genetics. Firstly, patients in genetics are often advised to recontact the clinician after several years for updated information. Secondly, a patient’s genetic makeup does not change over time in contrast to their physical appearance. This brings up issues of equity and justice. Would assertive patients who request reanalysis of their own accord have an advantage over patients who will not do this, or are not encouraged by their physician? And, subsequently, is there a responsibility for the laboratory in providing these equal opportunities?

Secondly, the literature shows that the option of systematic reanalysis is not yet captured in the informed consent procedures. This raises the question of whether a shift to a more dynamic form of consent is needed, one in which there is room to communicate new results at a later point in time as well as room for patients to (temporarily) withdraw their consent to be recontacted.

If it is decided to also include previous patients (or subgroups of these patients) in reanalysis, they also need to be recontacted for a consent update. This brings up new questions. Which kind of phenotypes should be included; should a selection be made based on phenotypes expected to give the greatest yield? And for how far back in time should recontacting be attempted? Should this be for all patients for whom NGS was performed? Or would this increase the workload too much and affect handling of new diagnostic questions, meaning that other criteria have to be applied?

Several articles showed that systematic reanalysis might be a cost-effective approach for improving diagnostic yield. However, the results here largely depend on practical considerations and trade-offs. For example, trio sequencing is shown to increase effectiveness, but samples from both parents are often difficult to acquire and therefore trio sequencing is not always feasible.

Finally, the effects on the current option of ad hoc reanalysis need to be considered when systematic reanalysis would be implemented. Would additional reanalysis on request at a certain point in time, for example because family members or other patients with a similar condition bring in new insights or a couple is planning a (next) pregnancy, remain practically possible and feasible, outside the systematic reanalysis intervals? And if not, what ethical and psychosocial issues will be at stake?

#### Points for consideration

For sustainable implementation of systematic reanalysis, different trade-offs should be considered in order to reach optimal results while minimizing the strain on resources and personnel. Decisions about these trade-offs will depend on the context and should be assessed for each setting.

Firstly, it should be assessed whether (systematic) reanalysis of stored genetic data is the optimal technique to increase diagnostic yield, or if retesting or reinterpretation only is sufficient, also depending on the situation or setting.

Secondly, the optimal approach needs to be considered. Partial manual reanalysis is much more labor-intensive but can also increase the diagnostic yield, whereas automated reanalysis is faster and requires less human capital but can also be less accurate. However, several promising automated approaches have already been described, including approaches using machine learning techniques [59, 69]. Moreover, automated approaches are shown to be effective for quick wins in diagnostic yield in recent publications [6, 72]. Clinical laboratories can therefore investigate whether an existing tool is suitable for implementation in their routine diagnostic and reanalysis process. Furthermore, guidelines for development and further implementation of these tools should be developed.

Thirdly, the methods included in systematic reanalysis need to be considered, as well as the information provided to the clinician and patient. For example, trio sequencing, after initial singleton sequencing, is shown to be more effective but will again increase the workload for the diagnostic laboratory, clinicians and even parents of patients.

Finally, a laboratory-initiated model of systematic reanalysis has been identified as an interesting option because the laboratory already has the (bio)materials and data for reanalysis available. This would remove the step in the process where the clinician or patient needs to request ad hoc reanalysis. Moreover, the laboratory is usually in charge of keeping analysis software up-to-date. However, the benefit of the clinician-initiated model is that the clinician has updated patient and family information. As laboratory staff have indicated that this information is crucial for analysis and reinterpretation, an automatic exchange of genotypic and phenotypic information might also be required.

For all these considerations, decisions about trade-offs need to be made that balance the added strains on resources while creating optimal results for (former) patients.

### Gaps, limitations and future research opportunities

Interestingly, only one of the four pillars of medical ethics was explicitly mentioned in the literature in the context of reanalysis (*beneficence*). The remaining three—*non-maleficence*, *autonomy* and *justice*—were described in the context of reinterpretation [13], but not yet for reanalysis, although these four principles together provide a framework for decision-making and should also be assessed in the context of decisions about implementing systematic reanalysis. For instance, assessing the significance of reanalysis in providing updated information to prevent or cease ineffective treatment approaches might effectively contribute to the practice of non-maleficence.

One of the main gaps in the current literature is the description of perceptions of GHPs regarding systematic reanalysis and the tools (to be) used for it. Evaluating these perceptions could provide valuable information regarding decision-making about the previously described trade-offs. In particular, the increasing availability of artificial intelligence-based tools demands different skills of the professionals involved and raises new questions regarding transparency and trustworthiness. Qualitative studies are thus needed to get more insight into this topic.

Furthermore, the perspectives of clinicians and patients, including previously undiagnosed patients (and their parents), need to be assessed with respect to their need for new information and its timing in order to design novel, flexible consent and recontact procedures.

Finally, for this scoping review articles were predominantly sourced from PubMed. Although a quick search in other databases such as Scopus didn’t initially yield in additional literature, articles addressing ELSI topics might have been missed. Therefore, a future literature study might benefit from expanding the search to other databases.

### Conclusions

We reviewed the current literature regarding systematic reanalysis of the NGS data generated in diagnostic genetic laboratories, focusing on ELSI aspects. Although systematic reanalysis is increasingly advised as an effective approach to increase diagnostic yield, questions regarding ELSI aspects come into play, and recognition of and reflection on these aspects is still lacking in current scientific literature. With this scoping review we have provided points for consideration for implementation of systematic reanalysis.

### Supplementary information

Supplementary table 1
